# On the Functions of the Lungs

**Published:** 1804-01-01

**Authors:** 


### On the Functions of the Lungs; *communicated by our Correspondent at Paris.*

A Paper has been read at the Medical Society at Montpelier, which has for its object to prove, that the function of the lungs is not to generate heat nor to carry off heat, as has been supposed, but for the purpose of introducing oxygen. The author, Dr. Thomas, considers the experiments of Godwin sufficiently accurate; and he says, that according to them, the quantity of heat combined with oxygen, taken in by respiration, is too small to account for the temperature of animals; he thinks that we should ascertain well before we determine how this heat, which may be disengaged from the oxygen inspired, is distributed through the body, to be certain that the caloric does not accompany the oxygen into the body. But let us suppose, says the author, that the oxygen does disengage its heat at the moment of fixing in the lungs, what should be the consequence?

1st. Says he, the veinous blood does not contain carbonic bonic acid in a state of gas; but he maintains that it does exist in a fixed state; the heat therefore necessary to give this carbon a gaeous form, must be taken into the account.

2d. Expiration carries off a quantity of vapour, but he does not admit that this vapour is the result of a combination in the lungs, for besides the difficulty of assigning the source of the hydrogen, there would be a still greater difficulty to account for that of oxygen, which we know to be in the proportion of 85 to 15. He does not speak of the consequences of that great degree of heat which must arise from the combustion of such a quantity of oxygen. He considers the evaporation from the lungs to be in itself more than enough to consume all the heat that could be disengaged from fifteen parts of oxygen; but as well as the heat necessary to carry off the humidity of the lungs, the cold air taken in, absorbs a considerable quantity.

3d. It is impossible to conceive that when we confine 200 cubic inches of atmospheric air, according to the calculation of Godwin, so small a degree of heat should be develloped; or when we inspire pure oxygen, that the only sensation we perceive is that of a cooling, refreshing feel. And further, observes the author, if chemical effects out of the body can be compared to what takes place within, we may find an analogous circumstance in support of the opinion advanced. Oxygen, says he, brought in contact with blood in a vessel, produces no sensible heat. Analogy would furnish many other reasons if they were admissible in an inquiry of this nature. The author brings many arguments, unnecessary to notice, in order to prove that the lungs are not intended as a refrigerating organ to the system. To determine the use of the lungs two considerations present themselves:

First.—The author thinks that the lungs may be useful in disengaging the blood of carbon; but this principle does not present itself pure in the blood in the lungs, for as there are 11 out of 100 parts of this acid in the air expired, and as carbon in acidifying absorbs twice its weight of oxygen, the inspired oxygen would be insufficient, even supposing that it acted all on the carbon: the same objections hold against carbon being dissolved in hydrogen. The author looks on carbon in the blood to be in an acid state, not in the state of oxyd, as M. Chaussier found that the oxyd of carbon gave a red colour to the blood.

A second effect, less equivocal than the former, is that produced by oxygen on the blood in the lungs; the author mentions mentions the many proofs of this fact, which are within the knowledge of most physiologists. The use of the introduction of this principle, he considers to be principally intended for revivifying the actions of the animals. Birds that take long flight, and require great exertion, take into their capacious lungs great stores of air; many insects, whose temperature is low, could not require for the support of their heat so extensive an apparatus as they have.

And lastly, the author observes, that the extent of the lungs in insects will be found to bear a very accurate proportion to the quantity of activity which they enjoy.

The author concludes by the following corrollaries:—

1st. Respiration cannot be assimilated to ordinary combustion, it is a real oxydation.

2d. The object of this function cannot be to support animal heat.

3d. The cause of animal heat is owing to a function purely vital.

4th. It is not to cool the body.

5th. The disengaging a certain portion of carbonic acid, the fixation of oxygen in the blood, and the support of irritability, are the direct effects of respiration.

